# Should Physicians Be Illiterate?

**Published:** 1890-05

**Authors:** 


### Should Physicians Be Illiterate?

During the session of the Legislature of New York for 1889, an act was passed providing that all persons intending to pursue the study of medicine must pass a preliminary examination in arithmetic, grammar, geography, orthography, American history, English composition, and the elements of natural philosophy. The act further provides that this examination should be conducted under the authority of the regents of the university, and in accordance with their rules, and that only those persons who have received a baccalaureate degree in course from a college or university, duly authorized to confer the same, shall be exempt from this preliminary examination. This enactment, and every movement in the same direction, is a source of great gratification to all who have recognized the low standard of attainments which many medical schools in this country have been disposed to encourage, and who are in favor of elevating the standard of medical attainments to a respectable plane, but, notwithstanding this enactment there is now cause for alarm in the fact that antagonism to this movement is being exhibited, and that too, by those in connection with medical institutions from which better things would be expected.

Some four or five years ago one of the most prominent medical institutions of this country established a rule requiring a preliminary education, and after testing the matter for a year or two, found that their classes were reduced in numbers from the fact that many who applied could not pass a satisfactory preliminary examination; and so the rule was reconsidered, and the requirement abandoned. This was a matter of sincere regret to all who have at heart the elevation of medical education. In opposition to this recent enactment, as well as that experiment to which reference has just been made, we can come to no other conclusion than this: that it is based upon financial consideration. It is a matter to be regretted that medical and dental colleges are in such position that the educational work must be influenced by large numbers in the classes and the money accruing from such enlarged numbers. The rush and scramble in many institutions of this kind is simply to secure the largest number of attendants and the largest income.

There is encouragement, however, in the fact that there is a movement on the part of many medical and dental institutions in our country looking toward a better condition of things. The lengthening of the term of instruction looks to a betterment, and there is with many institutions closer discrimination being exercised in regard to those who are accepted as students, and the importance of a preliminary examination is being more and more fully recognized and many are adopting it. It is to be hoped that the result will be that those who will in the future seek to enter our colleges will prepare themselves in a proper manner, with regard to a general education, that will enable them to carry forward the work upon which they propose to enter in a satisfactory and effective way.

